# Islets Transplantation at a Crossroads - Need for Urgent Regulatory Update in the United States: Perspective Presented During the Scientific Sessions 2021 at the American Diabetes Association Congress

**DOI:** 10.3389/fendo.2021.789526

**Published:** 2022-01-06

**Authors:** Piotr Witkowski, Louis H. Philipson, John B. Buse, R. Paul Robertson, Rodolfo Alejandro, Melena D. Bellin, Fouad Kandeel, David Baidal, Jason L. Gaglia, Andrew M. Posselt, Roi Anteby, Piotr J. Bachul, Yaser Al-Salmay, Kumar Jayant, Angelica Perez-Gutierrez, Rolf N. Barth, John J. Fung, Camillo Ricordi

**Affiliations:** ^1^ Transplantation Institute, Department of Surgery, University of Chicago, Chicago, IL, United States; ^2^ Section of Endocrinology, Diabetes & Metabolism, Department of Medicine, University of Chicago, Chicago, IL, United States; ^3^ Kovler Diabetes Center, University of Chicago, Chicago, IL, United States; ^4^ Division of Endocrinology, Department of Medicine, University of North Carolina, Chapel Hill, NC, United States; ^5^ Division of Endocrinology and Metabolism, Department of Internal Medicine, University of Washington, Seattle, WA, United States; ^6^ Diabetes Research Institute and Cell Transplant Center, University of Miami, Miami, FL, United States; ^7^ Department of Pediatrics, Schulze Diabetes Institute, Department of Surgery, University of Minnesota, Minneapolis, MN, United States; ^8^ Department of Translational Research and Cellular Therapeutics, Diabetes and Metabolism Research Institute, Beckman Research Institute of City of Hope, Duarte, CA, United States; ^9^ Joslin Diabetes Center, Harvard Medical School, Boston, MA, United States; ^10^ Division of Transplantation, Department of Surgery, University of California San Francisco, San Francisco, CA, United States; ^11^ Harvard School of Public Health, Harvard University, Boston, MA, United States; ^12^ Department of Surgery and Cancer, Faculty of Medicine, Imperial College London, London, United Kingdom

**Keywords:** islets transplantation, Food and Drug Administration (FDA), biological license application, regulations and policy, type 1 diabetes mellitus

## Abstract

Clinical islet allotransplantation has been successfully regulated as tissue/organ for transplantation in number of countries and is recognized as a safe and efficacious therapy for selected patients with type 1 diabetes mellitus. However, in the United States, the FDA considers pancreatic islets as a biologic drug, and islet transplantation has not yet shifted from the experimental to the clinical arena for last 20 years. In order to transplant islets, the FDA requires a valid Biological License Application (BLA) in place. The BLA process is costly and lengthy. However, despite the application of drug manufacturing technology and regulations, the final islet product sterility and potency cannot be confirmed, even when islets meet all the predetermined release criteria. Therefore, further regulation of islets as drugs is obsolete and will continue to hinder clinical application of islet transplantation in the US. The Organ Procurement and Transplantation Network together with the United Network for Organ Sharing have developed separately from the FDA and BLA regulatory framework for human organs under the Human Resources & Services Administration to assure safety and efficacy of transplantation. Based on similar biologic characteristics of islets and human organs, we propose inclusion of islets into the existing regulatory framework for organs for transplantation, along with continued FDA oversight for islet processing, as it is for other cell/tissue products exempt from BLA. This approach would reassure islet quality, efficacy and access for Americans with diabetes to this effective procedure.

## Introduction

Islets used for clinical allotransplantation have been successfully regulated as tissue/organs for transplantation in several countries and are recognized worldwide as a safe and efficacious therapy for selected patients with type 1 diabetes mellitus (T1DM) (1, 2). In contrast, in the United States (US), the Food and Drug Administration (FDA) considers pancreatic islets as biologic drugs and their use for treatment for diabetes has not yet shifted from the experimental to the clinical for last 20 years (1–3).

A recent FDA’s analysis of data submitted by the sponsor to support the BLA, uncovered in our opinion, major deficiencies in current regulations of human islets of deeply concern regarding patient safety and efficacy (4, 5). In contrast to other approved biologics and cell therapies, and despite application of drug manufacturing principles, technology, and regulations, the sterility and potency of islets cannot be verified prior to clinical use. This is inconsistent with the principles of the drug safety and efficacy regulatory system developed by the FDA. Yet, the FDA continues to demand that islets be regulated as drugs. This position has halted the development of the field over the last decade in the US, in contrast to other countries worldwide. The purpose of this manuscript was to shed more light on the islet and organ transplantation regulations in the US mostly unknown to the medical community and to propose an appropriate regulatory update urgently necessary for the field for safe clinical adoption of islet transplantation in the US.

## Regulation of Islets as Drugs in the US

Every new pharmacological and biologic drug requires a thorough safety and efficacy verification before being approved for use in clinical practice by the FDA. Biologic drug manufacturers/sponsors need to submit a BLA to the FDA for review and approval before commercial drug distribution. The BLA needs to prove: 1) safety and effectiveness of the drug based on preclinical and clinical studies; 2) appropriate labeling; and 3) consistent manufacturing method which provides a specific drug with specific characteristics that correlate with the desired clinical outcome.

**Figure 1:**
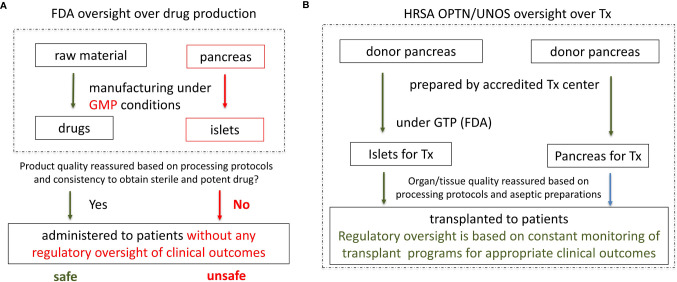
Current regulation of islet as drugs by the FDA and proposed regulation of islets as organs under HRSA, with aseptic islet processing under FDA’s Good Tissue Practice (GTP). **(A)** Current regulation of islet as drugs by FDA. Consistent quality of a drug and appropriate clinical effect are reassured based on: 1) consistency in manufacturing (processing protocols); 2) consistency in obtaining a sterile and potent product; 3) No clinical oversight is necessary. However, since islet sterility and potency cannot be consistently obtained and verified, quality of the islets and clinical outcome are uncertain. Without clinical oversight patient safety is in jeopardy. **(B)** Proposed regulation of islets as organs under HRSA with aseptic islet processing under FDA’s Good Manufacture Practices. Since sterility and potency of organs and islets cannot be objectively verified prior to transplantation, consistency and quality of organ/islet transplantation is reassured based on meeting the conditions for UNOS/OPTN accreditation, particularly: 1) implementation of the appropriate donor/organ processing protocols. 2) implantation of the appropriate islets aseptic processing protocols under FDA’s GTP. 3) close monitoring of the clinical outcomes (expected clinical outcomes warrant maintenance of the UNOS/OPTN accreditation).

Therefore, providing appropriate drug quality based on the drug identity, strength, purity and potency is a cornerstone for drug quality assurance (3). Confirmation of these quality attributes in the final drug assures safety and effectiveness of the clinical application (**Figure 1A**). The described quality assessment system works well for new drugs created from a standardized source of pharmacological material or from single cell products that are manipulated during the manufacturing process to develop new specific biological characteristics responsible for desired clinical outcome.

However, such quality assessments are ineffective, thus inappropriate for human organs used for organ transplantation (4, 5). Human organs including islets are not created in a laboratory or factory, but rather naturally developed in the human body with wide variability of characteristics, especially when retrieved from a deceased donor. Therefore, the application of drug regulations and quality testing is ineffective in verification of the appropriate quality of human organs as their characteristics are inconsistent. Also, if human organs for transplantation were regulated as drugs, human organs would not meet BLA requirements. Yet, human organs and islets save lives. Despite meeting the first two criteria for BLA approval (safety and effectiveness, and proper labeling), organs for transplant do not meet the 3^rd^ criterion for the BLA approval for drugs. Human organs are highly variable and their appropriate characteristics for required clinical outcome cannot be defined and verified prior to transplantation. Since human islets are effectively small complex organs, the same is true for them, and application of drug regulations cannot ensure islet quality (4–8) (**Figure 1A**).

Sterility is a basic requirement for any drug to be introduced to the patient intravenously. Because most cell based biological products can be maintained in the long-term product storage (e.g. in frozen form), there is sufficient time to test a sample of the final product for sterility in culture prior to clinical applications. In contrast, the sterility of human organs cannot be confirmed prior to transplantation, as there is not enough time prior to transplantation for comprehensive *ex vivo* organ testing. Similar to solid organs, human islets (as small organs) cannot be frozen or stored and effectively tested for sterility prior to clinical use. Instead, donor testing and diligence in aseptic organ and islet processing procedures are implemented to prevent recipient contamination.

Thus, human organs and islets require a different quality reassurance system than the FDA applies to drugs/biologics. Such regulatory framework has been developed and optimized over decades under HRSA, an agency of the Department of Health and Human Services (HHS); and successfully implemented by the Organ Procurement and Transplantation Network (OPTN) and the United Network for Organ Sharing (UNOS) (5) (**Figure 1B**).

## Human Organ Quality Assurance and Regulations In the US

Organ procurement organizations are not-for-profit and are accredited by the Center for Medicare and Medicaid Services (CMS) and UNOS to provide human organs, which meet appropriate safety and quality standards for clinical transplantation. In addition, the quality of human organs for transplantation is assured by transplant programs by continuous oversight of every element of the organ transplantation procedure. Transplant programs closely oversee and take the ultimate responsibility for every step of the transplant procedure starting with the selection of the organ donor and recipient, aseptic organ procurement, shipping, preparation and the transplantation procedure, as well as complex recipient medical care and final clinical outcomes. Full control over all elements of organ transplantation by the accredited transplant programs is a cornerstone of the human organ quality assurance by UNOS and OPTN (3) (**Figure 1B**).

In 1984, the US Congress approved the National Organ Transplantation Act (NOTA), which defined the human organ, its distribution, regulation and protection from commercialization (5, 9). Only OPTN/UNOS accredited transplant programs can transplant human organs. OPTN/UNOS accreditation is based on the implementation of the appropriate infrastructure, systems, procedures, protocols and personnel, which assure patient safety and effectives of the transplant procedures. This includes appropriate infrastructure, qualified personnel, clinical protocols, standard operating procedures (SOPs), and quality control and assurance (QC/QA) systems. Importantly, transplant programs are additionally scrutinized by OPTN/UNOS and the maintenance of UNOS accreditation is contingent on extensive reporting and provision of expected clinical outcomes as the ultimate measure of the quality of the medical care provided. Clinical outcomes are also scrutinized by insurance companies, which can limit contracting with transplant program for transplant procedures, if outcomes are worse than expected. There is also public scrutiny of clinical outcomes reported on the UNOS website for each individual transplant program.

Although the US Congress approved an amendment to NOTA in 1988, which incorporated subparts of human organs into the definition of human organ, the executive definition of human organ under OPTN has never been updated to include human islets as subparts of human pancreas (9). Other regulatory updates were passed by the Secretary of HHS, including the incorporation of blood vessels and vascularized composite allografts into the OPTN definition of human organs in 2007 and 2013, respectively (2). Unfortunately, human islets continue to be regulated with all other non-organ tissues and cells under the FDA, and are classified as human cells, tissues products (HCTPs).

However, in contrast to islets, those HCTP products (for example stem cell- derived differentiated cells) are *ex vivo* manufactured or substantially manipulated, can be frozen, stored, banked and fully characterized to reassure consistent sterility and potency of the product.

Human islets closely resemble small organs and vascularized composite allografts, as they also need to be retrieved fresh from cadavers, cannot be frozen or stored in tissue banks, cannot be or fully tested for sterility and potency, but still maintain their natural biological characteristics and need to be transplanted within hours into patients who require the same complex immunosuppressive medications as other organ transplant recipients.

**Figure 2:**
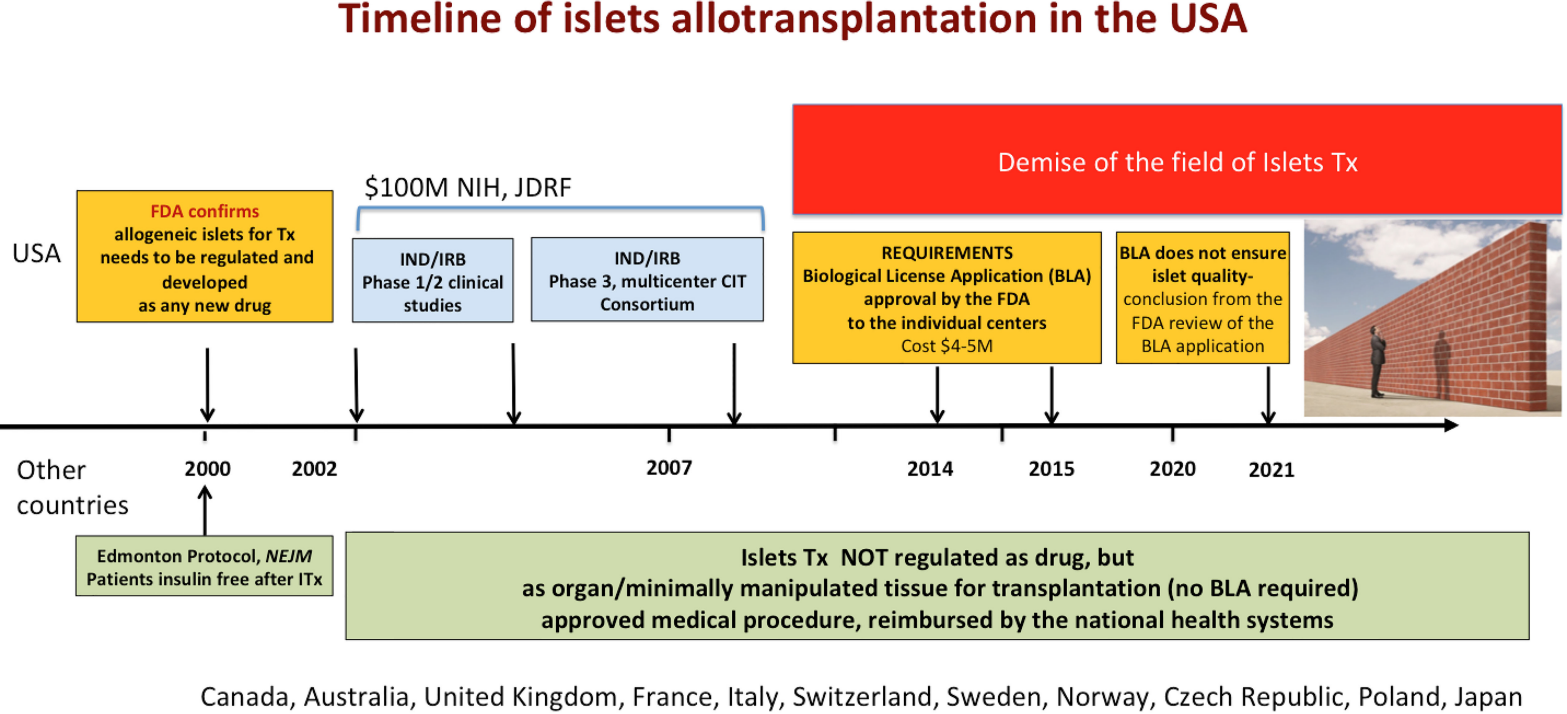
Timeline of the development of islet transplantation in the US and other countries. Part of the figure over the timeline depicts development of islet transplantation in the US. In the US, FDA has been regulating islets as biological drugs, which required clinical trials (performed from 2002 to 2014), as well as subsequent approval of the Biological License Application submitted by the sponsor. A recent review of the BLA application by the FDA concluded that despite application of the drug manufacturing technology and regulations, the quality and sterility of the islets cannot not be confirmed based on the release criteria. If the BLA is granted, the private BLA holder will obtain license to distribute islets of unknown quality, which could compromise patient safety. If the BLA cannot be granted due to organ like nature of the islets, there is no a path forward for islet transplantation in the US. Below the timeline, the status of islet transplantation in several other countries is presented. Islets are not regulated as drugs, but as organs/tissue for transplantation in these countries. Islet transplantation is an approved procedure, reimbursed by the national health systems in these countries.

In 2000, after the publication of break-through results of islet transplantation in Canada, the FDA reminded physicians in the US that they cannot just start transplanting islets as other organs, but rather need to follow regulations established for drug development, including BLA approval before standard of care clinical use (3). To address this issue, multicenter trials sponsored by the National Institutes of Health (NIH), JDRF and other foundations were performed over the next 14 years, confirming the safety, efficacy and reproducibility of the islet transplant procedure (2) (**Figure 2**). Unfortunately, none of the US academic medical centers has subsequently been able to submit a BLA for allogeneic islets, as they are not structured to act as pharmaceutical companies manufacturing drugs (2). Consequently, allogeneic islet transplantation is not approved for clinical use outside clinical trials, it is not standard of care treatment, and insurance carriers in the US do not reimburse it. As a result, activity in this field has gradually declined and after over 20 years of testing and over a hundred million dollars spent on pre-clinical and clinical studies islet transplantation is still not broadly available to Americans with T1DM (2).

Recently, a private company submitted a BLA for allogeneic islets transplantation in patients with brittle T1DM. The BLA is currently under the review by the FDA (**Figure 2**). If the BLA is approved, only those islets provided exclusively by that commercial company will be reimbursed by insurance (2).

In stark contrast, outside the US, allogeneic islets are successfully regulated as organs/tissues for transplantation (not as drugs) and are recognized as a standard of care reimbursed by national health systems in Canada, Australia and several European countries. Last year, France and Japan also announced exemption of islets from drug regulations based on up-to-date scientific information, which strengthens the worldwide consensus. Over 700 islet transplants have been successfully performed at University of Alberta in Canada within last 20 years. Importantly, this approach protects islets as other organs from commercialization for ethical reasons, which is not the case in the US (9).

## Based on Current Regulations the BLA for Allogeneic Islets Should Not Be Approved by the FDA

The FDA’s Advisory Committee met on April 15th, 2021, to provide feedback on data submitted in BLA the for allogenic islet transplantation. The advisors voted “yes” to the FDA’s question regarding whether islet transplantation is safe and effective (12 to 4, with one abstention), thus confirming that islet transplantation has overall favorable benefit: risk ratio for selected patients with T1DM (4).

However, as described above, for BLA approval the application must also prove that the implemented manufacturing methods consistently provide an islet product with specific biological characteristics, which correlate with appropriate clinical outcome. An FDA official, Dr. Sukhanya Jayachandra, presented during the meeting the results of an FDA review of the CMC (chemistry, manufacturing, controls) portions of the BLA, concluding that: “…the critical quality attributes (purity and potency) of the islets did not correlate with clinical effectiveness, and may not adequately evaluate lot-to-lot manufacturing consistency”. Clearly, islets do not meet this important criterion.

In other words, Dr. Jayachandra’s statement further supports the fact that it is not possible to determine whether isolated islets have the appropriate quality to provide expected therapeutic effect to the patient after transplantation despite applying drug regulations and testing (4).

In addition, the sterility of the islet product cannot be confirmed prior to transplantation by a standard testing in culture. Since islets are considered as a drug for intravenous infusion, commercial distribution of islets, which sterility cannot be reassured prior to use, is very concerning for patient safety.

Altogether, the quality of the islets required for the desired clinical effect cannot be confirmed prior to transplantation, and in such a situation granting BLA approval to the islet manufacturer would provide false reassurance to the patients and physicians about the quality and sterility of the offered islets. A for-profit company holding BLA would obtain permission to market and sell human islets despite not being able to verify quality and sterility before transplantation. Moreover, transplant centers, which are not able to obtain their own BLA will have no choice but to purchase human islets of unknown and unverifiable quality from a private company and transplant these into their patients. In our opinion, this results in an unethical situation, which compromises patient safety and trust in regulations and differers significantly from rules that governs organ transplantation. An islet product that does not meet criteria set forth in the BLA should not be approved under this regulatory framework.

## Islets Should Not Be Regulated as Drugs in US

Based on the current body of evidence and years of clinical research, islets should not be regulated as drugs. As described above, after 20 years of clinical testing as required by FDA, it has been shown that the application of the drug manufacturing [Good Manufacture Practice (GMP)] protocols and BLA requirements fail to assure appropriate human islet quality that correlates with patient safety and clinical effectiveness.

In addition to the FDA’s own report and arguments above, there is large amount of clinical data collected by the Collaborative Islet Transplant Registry (CITR) over the last 20 years from over 2,000 islet transplant procedures in the US and other countries which confirms the safety and effectiveness of islet transplantation without the need for BLA approval and drug related regulations. Therefore, islets should not be regulated as biologics; especially since the requirement of the BLA and drug regulation have been the main obstacle to clinical implementation of islet transplantation as a standard of care for patients with T1DM in the US. Allogenic islets should be exempt from BLA requirement as autologous islets have been for decades (2).

## Islet for US Collaborative

As leaders in the field of islet transplantation, we repeatedly requested the regulatory updates and re-evaluation of the need for BLA from the FDA and HHS. A working group of over 40 leaders and experts in the field as Islets for US Collaborative (www.isletsforus.org) performed a detailed analysis and published several articles to provide scientific evidence and promote the need for regulatory updates for allogeneic islets to the regulatory authorities in the US (1, 2, 4, 5). However, the FDA maintains its rigid position regarding BLA requirements for allogeneic islets. Our position against BLA requirements was also presented during the Open Public Hearing Session of the FDA’s Advisory Committee Meeting on April 15^th^, 2021 and was subsequently published (5).

## Summary of Arguments Supporting Islets to be Regulated as Any Other Organ/Tissue for Transplantation

1) Islets are human micro-organs and should thus be regulated along with other human organs, which are not regulated by the FDA, and for which a BLA is not required (5–8). Islets are comprise of many different types of cells with well-integrated functions, have their own internal vascular and neural network Islets maintain their own morphology and structure during processing and in the recipient after the transplantation. Furthermore, islets cannot be effectivelly cryopreserved, can only be maintained *in vitro* for short periods of time. Safe and effective islet transplantation require constant supervision by the transplant team starting with donor selection and continuing with post-transplant patient care in order to assure satisfactory clinical outcome of the transplant procedure.

2) Regulation of islets as organs under the HRSA is safer and more efficient than under the FDA.

HRSA has developed regulations to ensure the safe and ethical allocation and transplantation of human organs. Under HRSA, OPTN and UNOS oversee transplant programs that provide this treatment through a multidisciplinary team of transplant physicians. Thus, the UNOS/OPTN oversight framework is critical to maintain patient safety and effectiveness of this very complex therapy, which extends well beyond the transplant procedure. Islets considered as drugs under FDA oversight will not be subject to the comprehensive ongoing OPTN/UNOS oversight, and recipients of islets from a commercial BLA-holder, will not be subject to OPTN/UNOS post-transplant monitoring of patient outcomes (5). Islets will be less regulated than all other human organs. Regulating islets as drugs subject to a BLA, results in removal of the critical safeguards applied by OPTN/UNOS, which will jeopardize the safety of our patients.

3) The proposed regulatory adjustment can be implemented without changing FDA regulations for cell/tissue therapies (9).

The Secretary of HHS has the authority to designate allogeneic islets for transplantation as human organs under the OPTN Final Rule. Legally, it would conform to the statutory definition of the human organ under the National Organ Transplantation Act (NOTA 1988). This decision would provide OPTN/UNOS with legal authority for holistic, systematic clinical oversight over islet transplantation and would protect patients by ensuring the safety and efficacy of islet transplantation therapy. This would also prevent commercialization of human islets, which is prohibited under NOTA.

Health and Human Services’ (HHS) decision in 2013 to include vascularized composite allografts (VCAs) under OPTN/UNOS jurisdiction provides a strong precedent for including human islets under the OPTN final rule (2).

We do believe that proposed adjustments would not compromise islet processing regulatory oversight, which could remain subject to FDA Good Tissue Practice (GTP) requirements, since it has been successfully in place for over 30 years for the same human islets that are prepared for autologous use. The processing of the islets is exactly the same for autologous use as for allogeneic use (the same technology, reagents, equipment, personnel, facility).

4) Positive downstream effects of the inclusion of human islets into the definition of human organs under the OPTN Final Rule.

(a) Rapid implementation of islet transplantation as a standard of care; (b) Standard of care approval would lead to procedure reimbursement in accredited transplant centers; (c) Processing of islets would take place in multiple independent transplant centers, which would stimulate positive competition, promote cost-effectiveness, and improve access to the procedure for diabetic patients; (d) Unnecessary costs of drug related regulations would be avoided, making the procedure more affordable; (e) Reimbursement of the procedure would offset of the cost of research, which would stimulate progress in the field; (f) Progress in islet transplantation could stimulate progress in other beta cell replacement therapies and in regenerative medicine.

In conclusion we hope that our voice and arguments will be heard and taken into considerations by the FDA, HRSA and HHS to update the regulations regarding allogeneic islet for transplantation. It is critical for the sake of our patients and for the future of the islets transplantation in the US.

## Data Availability Statement

The original contributions presented in the study are included in the article/supplementary material. Further inquiries can be directed to the corresponding author.

## Author Contributions

PW, LP, JB, PR, RA, AP, MB, and CR contributed to conception and design of the study. RoiA, PB, YA-S, KJ, and AP-G collected data. PW, RoiA, and PB wrote the first draft of the manuscript. All authors contributed to manuscript revision, read, and approved the submitted version.

## Funding

PW and LHP were supported by the NIDDK P30 DK020595 grant and the Kovler Family Fund.

## Conflict of Interest

The authors declare that the research was conducted in the absence of any commercial or financial relationships that could be construed as a potential conflict of interest.

## Publisher’s Note

All claims expressed in this article are solely those of the authors and do not necessarily represent those of their affiliated organizations, or those of the publisher, the editors and the reviewers. Any product that may be evaluated in this article, or claim that may be made by its manufacturer, is not guaranteed or endorsed by the publisher.
